# Efficacy of oral afoxolaner and a combination of afoxolaner, moxidectin, and pyrantel against *Amblyomma maculatum* in dogs

**DOI:** 10.1186/s13071-025-07186-z

**Published:** 2025-12-18

**Authors:** Joseph Prullage, Jeffrey Shryock, Pascal Dumont, Liezl Whitehead, Stephen Yoon, Ricarda Süssenberger

**Affiliations:** 1 Boehringer Ingelheim Animal Health, Missouri Research Center, 6498 Jade Rd, Fulton, MO 65251 USA; 2 Boehringer Ingelheim Animal Health, Georgia Research Center, 1730 Olympic Dr, Athens, GA 30601 USA; 3 Clinvet International (Pty) Ltd, Uitzich Road, Bainsvlei, Bloemfontein, 9338 South Africa; 4 Boehringer Ingelheim Vetmedica GmbH, Binger Str. 173, 55216 Ingelheim Am Rhein, Germany

**Keywords:** Canine, Afoxolaner, *Amblyomma maculatum*, Treatment, Control

## Abstract

**Background:**

*Amblyomma maculatum* is a tick with a broad host range that is undergoing an expansion of its range within the USA. When feeding the predilection sites on the host are the head and ears and due to the long mouthparts, it can cause significant lesions that can lead to infection. It has also been implicated as a vector of *Hepatozoon americanum*, the causative agent of canine hepatozoonosis and a spotted fever group *Rickettsia*, *Rickettsia parkeri*.

**Methods:**

Two randomized, blinded, negative controlled studies were conducted to determine whether treatment with afoxolaner (NexGard^®^, Boehringer Ingelheim) or a combination of afoxolaner, moxidectin, and pyrantel (NexGard^®^ Plus, Boehringer Ingelheim) effectively treats and controls infestations of *A. maculatum* on dogs. For each study, ten healthy dogs were randomly assigned to each treatment group. In one study there were three treatment groups: an untreated control, NexGard^®^-treated group, and NexGard^®^ Plus-treated group. The other study had an untreated control group and a NexGard^®^ Plus-treated group. Dogs were infested with approximately 50 unfed adult *A. maculatum* prior to treatment for evaluation of efficacy against existing infestations and then three times after treatment for evaluation of persistent efficacy. In each study the appropriate treatment groups were treated with either NexGard^®^ or NexGard^®^ Plus with afoxolaner targeted at 2.5 mg/kg, and ten control dogs were untreated. For evaluation of efficacy, live ticks were counted and removed from each dog at 72 h after treatment or subsequent infestations.

**Results:**

NexGard^®^ and NexGard^®^ Plus were > 99% effective against established infestations of *A. maculatum* compared with the control group (*P* < 0.0001). NexGard^®^ and NexGard^®^ Plus were ≥ 92% effective against reinfestation with *A. maculatum* through Day 31 of the studies (*P* < 0.0001).

**Conclusions:**

The results of these studies demonstrate that NexGard^®^ and NexGard^®^ Plus administered once at or near the minimum recommended dose of 2.5 mg/kg afoxolaner is effective for the treatment of existing *A. maculatum* infestations and for the control of infestations through Day 31.

**Graphical Abstract Created in BioRender. Morschhaeuser, B. (2026) https://BioRender.com/081ioe1:**

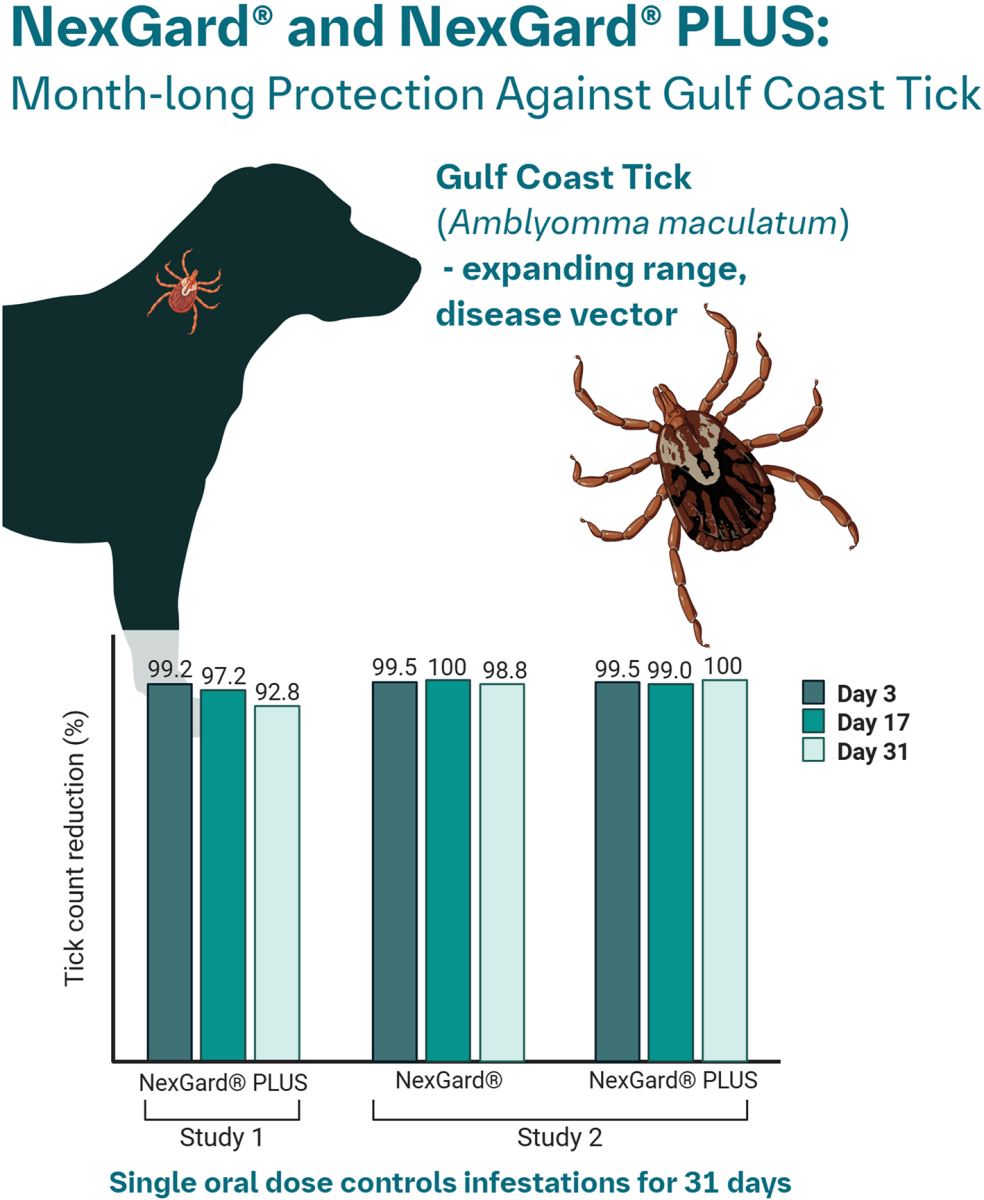

## Background

*Amblyomma maculatum*, commonly called the Gulf Coast tick, is an ixodid tick that acquired its name from its historic range along the Gulf and southern Atlantic coasts of the USA. Its range extends into Central and South America in countries that border the Gulf of Mexico and the Caribbean Sea [1]. It is commonly found in more xeric habitats than those typically occupied by the lone star tick, *Amblyomma americanum*, such as open fields, prairies, and coastal plains. Range expansions from the coastal regions into Oklahoma and Kansas probably occurred in the 1950s and 1960s [2]. More recently a range expansion into Arkansas, Virginia, Maryland, Delaware, and Kentucky have been reported [3–6]. Seasonal activity varies depending on the location of the population of *A. maculatum*. Larval and nymphal populations peak in January and February, respectively, and adult populations peak in September in Texas. Populations in Oklahoma and Kansas have peak populations approximately 5 months earlier for each life stage [7].

*Amblyomma maculatum* feeds on a wide range of hosts with immature stages infesting small mammals and birds and adult stages typically infesting a wide range of mammals, including dogs and cats [7]. The adult stage predilection sites for feeding are on the head and especially the ears of their host, resulting in aggregations of ticks, which can contribute to formation of lesions that then can lead to infection.

Similar to other ticks, *A. maculatum* is a vector of diseases, and for dogs the most serious of these is American canine hepatozoonosis (ACH) caused by *Hepatozoon americanum*. *Amblyomma maculatum* is the only known tick vector and definitive host of this disease and is infected while feeding as larvae or nymphs [8]. The infection can pass transstadially, but research on the closely related species *Hepatozoon canis* would indicate that transovarial passage does not occur [10, 11]. Transmission to dogs occurs by consumption of infected ticks during grooming or consuming infected prey [11, 12]. Diagnosis of *H. canis* is difficult, and therefore, the actual incidence of ACH is unknown [13, 14]. However, in a survey of 614 suspected cases in the USA, *H. americanum* was detected in states where *A. maculatum* has historically occurred, and in several of the states where *A. maculatum* has recently established, including Kentucky and Virginia [13]. Dogs may remain infected for greater than 5 years, but without treatment, most die within 1 year [14].

*Amblyomma maculatum* is also the vector of *Rickettsia parkeri*, a spotted fever group *Rickettsia* that, until 2004, when it was discovered to infect humans, was not differentiated from Rocky Mountain spotted fever (RMSF) [15]. *Rickettsia parkeri* causes a disease in humans that is milder than that seen with RMSF. In addition to humans, *R. parkeri* has been detected in dogs and the house mouse, *Mus musculus* [16, 17]. *Rickettsia parkeri* has been shown to be transmitted transstadially and transovarially in *A. maculatum,*, and it has been proposed that, by this means, *A. maculatum* serves as the reservoir host for this *Rickettsia* [18, 19].

The lesions caused by *A. maculatum* feeding on dogs and the transmission of *H. americanum* and *R. parkeri* demonstrate the importance of controlling infestations of this tick on dogs. This paper reports the results of two studies using afoxolaner (NexGard^®^, Boehringer Ingelheim) or a combination of afoxolaner, moxidectin, and pyrantel (NexGard^®^ Plus, Boehringer Ingelheim) for the treatment of existing infestations and the control of *A. maculatum* infesting treated dogs.

## Methods

Dogs were acclimated to the facilities for at least 7 days and managed similarly and with due regard for their well-being. The study design was reviewed and approved by the sponsor’s and local institutional animal care and use committees and met USDA-APHIS (US Department of Agriculture Animal & Plant Health Inspection Service) animal welfare requirements. The studies were conducted in accordance with Good Clinical Practices [20]. Standards included masking of personnel and randomization to treatment groups. A physical examination was conducted on Day −7 during acclimation to document the health and suitability of all animals for inclusion in the study, and dogs were observed for general health at least once daily throughout the study.

Two studies were conducted with the study designs outlined in Table 1. Healthy dogs were used in all studies and were not treated with any ectoparasiticide or long-acting parasiticide within 3 months of the start of the studies. For Study 1, 20 beagle and mongrel dogs, 1 male and 19 female, that were 12–86 months old were used. For Study 2, 30 beagle dogs, 14 male and 16 female, that were 8–13 months old were used.

**Table 1:** Study design and treatment details

Study 1 ^a^	Treatment group	Body weight range (kg)	Afoxolaner dose range (mg/kg)	Number of dogs
1	Untreated control	12.0–14.5	Not applicable	10
	NexGard^®^ Plus	10.4–14.6	2.60–2.96	10
2	Untreated control	6.9–10.8	Not applicable	10
	NexGard^®^	8.0–12.6	2.80–3.72	10
	NexGard^®^ Plus	7.9–10.8	2.53–3.65	10

^a^Study 1 was conducted in Bloemfontein, South Africa; Study 2 was conducted in Georgia, USA

Dogs were housed individually in stainless steel cages with epoxy-coated floors, assigned to each dog throughout the study, in compliance with local animal welfare regulations. Dogs had visual and auditory contact with conspecifics, but no physical contact between dogs was possible. Water was provided ad libitum either in water bowls or by automatic lickers. Feed was provided in bowls in sufficient quantity determined by age and growth requirements to fulfill nutrient and energy requirements and ensure the health and well-being of the dogs.

Dogs were infested with 50 (± 5) laboratory-reared *A. maculatum* (sex ratio, 1:1 male:female) on Day −7 or −6, and ticks were removed and counted 72 h later to establish that dogs included in the study could be adequately infested to evaluate effectiveness. In Studies 1 and 2, dogs were assigned to housing and to the treatments completely at random, with ten dogs assigned to each treatment group, as outlined in Table 1. The PLAN procedure of SAS Version 9 was used in all studies to assign dogs to treatments and cages.

Dogs were infested on Day −1 with 50 *A. maculatum* ticks (1:1 male:female). On Day 0, a combination of chews of the appropriate product were administered once to the dogs treated with NexGard^®^ (afoxolaner only; Boehringer Ingelheim) and NexGard^®^ Plus (afoxolaner, moxidectin, and pyrantel; Boehringer Ingelheim) in each study to provide a dose of afoxolaner, the acaricidal component, as close as possible to the minimum recommended dose of 2.5 mg/kg. In Study 1, dogs were fed approximately 60 min prior to treatment. In Study 2, the untreated control dogs and the dogs treated with NexGard^®^ Plus were fed 60 min prior to treatment, and the dogs treated with NexGard^®^ were fasted prior to treatment and were not offered food until after the 4-h post-treatment health observation. Protective clothing was changed, and tables were cleaned between the treatment of each dog to prevent cross-contamination. In both studies, animals were observed hourly for 6 h, then at 8 and 12 h, and at 24 h following treatment of the last animal to detect any adverse reaction to treatment.

All personnel involved with the evaluation of effectiveness (tick counts) and the recording of health observations were unaware of each animal’s treatment assignment.

For Study 1, dogs were infested with laboratory-reared *A. maculatum* that had been collected in Hutchinson, Reno County, Kansas, USA, in June 2021. For Study 2, dogs were infested with laboratory-reared *A. maculatum* that originally had been collected southeast of Goliad in Refugio County, Texas, in the mid-1980s. Additional progeny (from gravid females collected off grazing beef cattle at the same ranch location and from gravid females collected off livestock in Brazos County, Texas) were mixed into the colony, with the latest addition in 2023.

Infestation with *A. maculatum* after treatment on Days 14 and 28 was performed the same as with the pretreatment infestations. For both studies, dogs were sedated prior to infestation with ticks. For Study 1, medetomidine or dexmedetomidine was administered intramuscularly to produce mild sedation. Sedation was reversed using atipamezole. For Study 2, dogs were mildly sedated with acepromazine, administered orally. After sedation, dogs were placed in infestation chambers that permitted dogs to stand and lie comfortably, and the ticks were placed on the side of each dog in Study 1 and between the shoulder blades in Study 2. The dogs remained in the container for no longer than 5 h, during which their safety and comfort were monitored.

Tick counts after treatment with the study drugs were performed 72 h after treatment or 72 h after each subsequent infestation. Dogs were brought to the tick counting area in a unique randomized order for each tick count after Day 0. For counting, the hair coat was searched by parting the hair, ticks were removed, and the live or dead status was confirmed. Once the entire body of the animal had been examined for ticks, the animal was combed using a fine-toothed flea comb for secondary confirmation of tick removal. Protective clothing, gloves, forceps, and flea combs were changed or cleaned between each dog to prevent cross-contamination.

Efficacy with respect to live tick counts was calculated using the formula [(C − T)/C] × 100, where C is the arithmetic mean calculated from the least squares mean (LSMEAN) of the live tick counts for the control group and T is the arithmetic mean calculated from the LSMEAN for the appropriate treated group obtained from the MIXED procedure.

The counts of the live ticks after treatment or subsequent infestations of each treated group were compared with the counts of the untreated control group using an F-test at each time point separately. The MIXED procedure in SAS Version 9.4 was used for the analysis, with group listed as a fixed effect.

## Results and discussion

Dose ranges of afoxolaner, the acaricidal component of NexGard^®^ and NexGard^®^ Plus, are listed in Table 1, and all are in the bottom half of the 2.5–6.0 mg/kg dose range for NexGard^®^ and the 2.5–5.0 mg/kg dose range for NexGard^®^ Plus. The World Association for the Advancement of Veterinary Parasitology (WAAVP) guidelines for the evaluation of the efficacy of parasiticides for control of fleas and ticks state that a tick retention rate in the control group of 20% is generally acceptable to demonstrate that the tick attachment was acceptable [21]. In all of the studies reported here, nine or ten of the dogs in the control group had 24 or more ticks attached, i.e., 48% attachment rate. Tick counts for each of the post-treatment counts and analyses are presented in Table 2.

**Table 2:** Arithmetic mean and statistical analysis of live *A. maculatum* counts after one treatment with NexGard^®^ or NexGard^®^ Plus

Study^a^	Treatment group		Day 3	Day 17	Day 31
1	Untreated control	AM	38.2	36.0	33.4
		Range	29–50	30–44	29–40
	NexGard^®^ Plus	AM	0.3	1.0	2.4
		Range	0–2	0–10	0–8
		Percent efficacy	99.2	97.2	92.8
		ANOVA F-value	F_(1,18)_ = 271.76	F_(1,18)_ = 334.09	F_(1,18)_ = 458.10
		*P*-value	< 0.0001	< 0.0001	< 0.0001
2	Untreated control	AM	40.6	38.5	34.7
		Range	28–52	25–47	21–42
	NexGard^®^	AM	0.2	0.0	0.4
		Range	0–1	0–0	0–3
		Percent efficacy	99.5	100.0	98.8
		ANOVA F-value	F_(1,18)_ = 309.90	F_(1,18)_ = 278.79	F_(1,18)_ = 256.69
		*P*-value	< 0.0001	< 0.0001	< 0.0001
	NexGard^®^ Plus	AM	0.2	0.4	0.0
		Range	0–1	0–2	0–0
		Percent efficacy	99.5	99.0	100.0
		ANOVA F-value	F_(1,18)_ = 309.90	F_(1,18)_ = 269.43	F_(1,18)_ = 268.17
		*P*-value	< 0.0001	< 0.0001	< 0.0001

Arithmetic mean was calculated from the least square means of the appropriate treatment group. Percent efficacy = 100[(C − T)/C], where C and T are arithmetic means calculated from the least squares mean of the appropriate treatment group. *P*-values were two-sided *P*-values from the MIXED model on counts of the appropriate treatment group. AM, arithmetic mean; ANOVA, analysis of variance

^a^Study 1 was conducted in Bloemfontein, South Africa; Study 2 was conducted in Georgia, USA

In all studies, NexGard^®^ and NexGard^®^ Plus were > 99% effective against established infestations of *A. maculatum*. In Study 2, NexGard^®^ was ≥ 97% effective against reinfestation by *A. maculatum* through Day 31 when treated at or near the minimum dose of afoxolaner. In both studies, NexGard^®^ Plus was > 92% effective against reinfestation by *A. maculatum* when treated at or near the minimum dose of afoxolaner. The tick counts for the NexGard^®^- and NexGard^®^ Plus -treated groups were all significantly different from the control group (*P* < 0.0001).

No adverse events related to treatment were observed in any of the NexGard^®^ -treated dogs in Study 1. In Study 2, there was one instance of diarrhea in two dogs in the untreated control group, in one dog in the NexGard^®^-treated group, and in two NexGard^®^ Plus-treated dogs within 24 h of treatment. Due to the proximity to treatment, the instances of diarrhea in the NexGard^®^- and NexGard^®^ Plus-treated groups in Study 2 were possibly related to treatment.

Historically, the Gulf Coast tick had a rather restricted range; however, transportation of infested animals, movement of wildlife, habitat modification, and potentially climate change are resulting in an expansion of this range [9, 22]. In addition, studies demonstrating that *A. maculatum* is a vector of *H. americanum* and *R. parkeri* further underline the importance of this tick to the health of both dogs and humans. The results of these studies using two different isolates of *A. maculatum* demonstrate the efficacy of afoxolaner within 72 h against this tick in both established infestations and its sustained efficacy over a month after treatment. Afoxolaner in NexGard^®^ and NexGard^®^ Plus offers a convenient method for controlling *A. maculatum* on dogs for their health and comfort and potentially reducing contact between humans and this disease vector.

*Amblyomma* spp. ticks have been shown to be the dose-limiting tick for isoxazoline ectoparasiticides. This may be partly explained by the time it takes for these ticks to find a suitable feeding site. *Amblyomma* spp. ticks are hunting ticks; that is, they will move to acquire a host [23–26]. This is in contrast to other species of ticks such as *Dermacentor* spp. and *Ixodes* spp. that remain on vegetation and wait for a host to come into contact with them, also called ambush ticks [23, 27–29. This predilection for movement seems to carry over once *A. maculatum* and *A. americanum* ticks find a host, as they wander for several hours prior to starting attachment, as observed by one of the authors (J.P.). Therefore, a delayed start of feeding, compared with other tick species, is observed, which, together with *A. maculatum* being a slow feeder, results in tick efficacy evaluated at counting times of 72 h. Fluralaner is registered for control of *Dermacentor variabilis*, *Ixodes scapularis*, and *Rhipicephalus sanguineus* for 12 weeks and for control of *Amblyomma americanum* for 8 weeks [30]. A combination of sarolaner, moxidectin, and pyrantel is registered for control of *A. americanum* with efficacy demonstrated at 72 h after treatment or subsequent infestation, similar to NexGard^®^ and NexGard^®^ Plus. Three studies were conducted for the registration of sarolaner, moxidectin, and pyrantel against *A. maculatum*. In two of these studies, tick counts were conducted 48 h after treatment or infestation with efficacy ≥ 90% through day 28. Efficacy at Day 35 was 97.0% in one study and 76.3% in the second study [31]. Prior studies have shown that NexGard® and NexGard® Plus are effective for a month against *A. americanum* in contrast to results reported in a previous article [32, 33]. These studies provide further evidence that NexGard^®^ and NexGard^®^ Plus are effective against *Amblyomma* spp. ticks, specifically *A. americanum* and *A. maculatum*, for a full month.

## Conclusions

The results of these studies demonstrate that NexGard^®^ and NexGard^®^ Plus, administered at or near the minimum recommended dose of 2.5 mg/kg afoxolaner, are effective for the treatment of established infestations of *A. maculatum* and for the sustained control of this tick through Day 31 after a single treatment. These studies also provide further support for the month-long efficacy of NexGard^®^ and NexGard^®^ Plus against *Amblyomma* spp., specifically *A. americanum* and *A. maculatum*, ticks.

## Data Availability

All relevant data supporting the conclusions of this article are included within the article.

## Abbreviation

LSMEAN: Least squares mean
